# Spatiotemporal transcriptomic signatures of resident cardiac macrophages and macrophage-associated spatial states during acute myocardial infarction

**DOI:** 10.3389/fimmu.2026.1882796

**Published:** 2026-10-20

**Authors:** Jonathan Yap, Jason S. Irei, Tianmai Bishop, Selena Vanapruks, Hector A. Cabrera-Fuentes, William A. Boisvert

**Affiliations:** 1 Center for Cardiovascular Research, John A. Burns School of Medicine, University of Hawaii, Honolulu, HI, United States; 2 División de Estudios de Posgrado e Investigación, Tecnológico Nacional de México/Instituto Tecnológico de Tijuana, Tijuana, B.C, Mexico

**Keywords:** acute myocardial infarction (AMI), ischemia/reperfusion injury (IRI), NanoString/Visium analysis, resident cardiac macrophages (RCM) isolation, S100a9 macrophage-associated signaling, spatial transcriptomics

## Abstract

**Background:**

Acute myocardial infarction (AMI) followed by reperfusion result in a phasic response by cardiac macrophages. During the first 7 days after AMI, macrophage populations exhibit dynamic phenotypic changes. Ultimately, inflammatory damage results in ventricular remodeling and fibrosis. Macrophages are critical to cardiac function and long-term prognoses.

**Objective:**

Elucidate the spatiotemporal behavior of specific macrophage subtypes during AMI and identify candidate macrophage-associated transcriptional programs during early AMI.

**Methods:**

We implemented an ischemia/reperfusion injury (IRI) mouse model of AMI to assess cardiac macrophage behavior during AMI. Resident cardiac macrophages (RCM) were isolated using flow cytometry and analyzed with NanoString. Pathway enrichment analysis annotated statistically significant genes. We employed transcriptome-wide, spatiotemporal analysis of cardiac tissue samples during AMI.

**Results:**

NanoString analysis of pooled RCM samples identified candidate differentially expressed genes (DEGs) between time points, and pathway enrichment analysis suggested inflammatory, reparative, and extracellular matrix-associated programs during early AMI. Visium spatial transcriptomic analysis of cardiac tissue samples identified spatial patterns consistent with six macrophage-associated transcriptional states within the cardiac compartment. Integration of NanoString, Visium, and immunostaining data revealed temporal and spatial patterns of S100a9-associated macrophage signatures during early AMI. We also observed spatial co-localization of macrophage-associated and fibrosis-associated transcripts in injured regions.

**Conclusions:**

These findings provide insight into cardiac macrophage-associated transcriptional programs during AMI and identify candidate inflammatory, reparative, and fibrosis-associated pathways for future mechanistic investigation.

## Introduction

Inflammation contributes to the pathology of innumerable disease states and conditions. In the case of acute myocardial infarction (AMI), pro-inflammatory signaling initiates an irreversible cascade of cardiac responses to coronary ischemia. These physiological changes take place regardless of whether the tissues or vasculature are reperfused. Distressed myocardial tissues can only be resuscitated through timely reperfusion intervention which prevents further damage and cellular necrosis (1). Paradoxically, reperfusion of tissues results in myocardial ischemia/reperfusion injury (IRI). At present, it is generally understood that an AMI event following vascular occlusion and coronary ischemia proceeds in a phasic manner consisting of an inflammatory phase (0–48 hours), an anti-inflammatory/proliferative phase (3–4 days), and a reparative phase (≥7 days) (2, 3). As the predominant immune cell type in the cardiac compartment, macrophages are heavily involved as effector cells and regulators in each stage of AMI. Resident cardiac macrophages (RCM) are derived from yolk sac progenitor cells during embryonic development and are maintained as a self-renewing population in the steady state heart. Under homeostatic conditions, RCM are necessary for proper remodeling of the coronary plexus and maturation of coronary vasculature (4). In addition to cardiac development, RCM also participate in the AV node conduction and immunosurveillance which involves detection/phagocytosis of pathogens and efferocytosis of cell debris (5–8).

Ischemic conditions during AMI results in extensive cellular necrosis and damage to cardiac tissues. Such large-scale cell death triggers release of damage-associated molecular patterns (DAMPs). DAMP-induced signaling is often initiated by alarmin proteins, extracellular RNA, mitochondrial DNA and other cytokines (9, 10). Along with cardiomyocyte death, RCM populations are also significantly diminished during this period, thereby limiting their anti-inflammatory capacity. Replacement of RCM occurs by the rapid infiltration of monocyte-derived macrophages (MDM) as evidenced by elevated monocytopoiesis lasting several days after an AMI event (11). Experimental ablation of infiltrating MDM using diphtheria toxin or RNA interference results in the recruitment of fewer pro-inflammatory macrophages to the infarct zone and reduced cardiac fibrosis. Conversely, depletion of RCM leads to significantly increased fibrosis within cardiac tissues following AMI (4, 12). In addition to these findings, it is becoming clear that an appropriate balance of both pro-inflammatory and anti-inflammatory macrophages is required to properly regulate cardiac tissue repair as complete inhibition of pro-inflammatory macrophages is counterproductive to cardiac tissue repair (13). The robust immune response that initially occurs following AMI is hypothesized to facilitate efferocytosis and clearance of cell debris, both of which are critical to resolution of the inflammatory process (3). During the anti-inflammatory/proliferative stage of AMI, macrophage population consists almost entirely of MDM which begin to proliferate and polarize into reparative macrophage phenotypes. Intriguingly, in spite of their dichotomous origins, infiltrating MDM are able to recapitulate the functional repertoire of heterogeneous macrophage populations including RCM to a large degree (4). However, research has also shown that individuals with elevated MDM and those of advanced age tend more often to have a disproportionate ratio of RCM to MDM, in effect, increasing comorbidities, incidence of a second AMI event, and death (14, 15). Even in the reparative phase when wound healing and tissue remodeling take place, current research shows a subpopulation of fibrogenic macrophages. These MDM exhibit an anti-inflammatory phenotype and contribute to collagen formation and tissue repair following cardiac tissue damage (16, 17).

With this study, our overarching goal was to demonstrate the spatial distribution over time of important macrophage subtypes during the early stages of AMI. While single-cell transcriptomics is a valuable tool in stratifying populations of cell types, spatial transcriptomics allows us to visualize the tissue distribution of macrophage subtypes. This type of analysis provides perspectives into the prevalence and migration patterns of specific macrophage populations within the cardiac compartment. Studies utilizing this have demonstrated that S100a8 and S100a9 are important in localized inflammation, a finding which is consistent with our observations (18, 19). Studying primary RCM continues to be a challenging endeavor due to the relatively small number of these cells present in the murine heart. Flow cytometry used in tandem with NanoString technology allowed us to sort and analyze RCM using a robust multiplex transcriptional analysis of important genes involved in the immune response. RCM are of particular interest given their multifaceted functionality regarding the specific pro-inflammatory/anti-inflammatory gene expression patterns during the early stages of AMI. We also leverage our NanoString results by employing pathway analysis, thereby providing putative signaling pathways affected by this macrophage population.

In our work, we performed LAD ligation surgery in murine models as an effective means of initiating IRI to study the RCM-associated transcriptional changes during AMI. Moreover, we incorporated spatial transcriptomics which offers novel insights into macrophage function and phenotypic heterogeneity within the cardiac compartment. This approach enabled us to compare differentially expressed genes (DEG) and relative abundance within macrophage populations during the inflammatory and proliferative phases while simultaneously visualizing macrophage presence as it relates to the developing infarct.

## Methodology

### IRI surgery

Six to eight week old male C57BL/6 mice (Jackson Lab., Bar Harbor, ME) were anesthetized and a temporary ligation with a suture was applied to the left anterior descending coronary artery (LAD) (20, 21). After 30 minutes, the suture is removed allowing for reperfusion of ischemic cardiac tissue and initiating IRI. Animals were then euthanized and the hearts were isolated 2 days and 4 days post-injury for analysis. All studies were performed in accordance with the ethical guidelines and regulatory standards set by the University of Hawaii. Mice were induced with 5% isoflurane in oxygen at 1 L/min using a precision vaporizer. Adequate anesthesia depth was confirmed by toe and tail pinch. Anesthesia was maintained with 2–3% isoflurane during surgery. Mice were euthanized by CO_2_ exposure using a gradual-fill method at a displacement rate of 70% of the chamber volume per minute.

### Immunofluorescence staining of S100A9 and macrophages

Immunofluorescence staining was carried out using 10 µm serial cryosections of control mouse hearts and from each time point after MI. Primary antibodies for CD68 (Invitrogen 14-0681-82) and S100A9 (Invitrogen PA5-79950) were used at a working concentration of 1:50 and 1:300 respectively. Samples were blocked using 2% BSA for one hour. Incubation with primary antibodies was carried out overnight at 4°C. After washing, secondary antibodies; Donkey Anti-Rat IgG (H+L) Highly Cross-Adsorbed Secondary Antibody, Alexa Fluor 568 (ThermoFisher, A78946) and Goat Anti-Rabbit IgG (H+L) Highly Cross-Adsorbed Secondary Antibody, Alexa Fluor 568 (ThermoFisher, A-11036) were applied for one hour at room temperature and followed by addition of DAPI (1:100) for 5 minutes. Images were acquired using the Leica Thunder Live Cell 3D microscope available through the University of Hawaii Cancer Center, Microscopy, Imaging, and Flow Cytometry Equipment Core. Protein expression was quantified with the ImageJ software by isolating and measuring total pixel-area coverage (Px^2^) of the red channel as defined by the Alexa Fluor 568 emission spectra. Due to the extreme auto fluorescence of cardiac tissue in the green channel, selected images were pseudo-colored from red to green for visualization of colocalization.

### Tissue digestion

Following IRI surgery, hearts were removed and perfused with 15 mL cold DBPS + 0.9mM CaCl_2_ solution using a 20 mL syringe fitted with a 25G needle then placed in individual wells of a 12-well plate containing 5 mL enzyme digestion buffer per 100 mg of heart tissue. Digestion buffer consisted of 600 U/mL collagenase IV, and 1.2 U/mL dispase in 25 mL of DBPS + 0.9mM CaCl_2_ solution. Whole hearts were then transferred into 6 cm Petri dishes where they are minced into a slurry using dissecting scissors. The tissue slurry was allowed to digest for an additional 15 minutes at 37 °C. The contents of each well were passed through a 40 µm filter before being centrifuged for 15 minutes at 200 x g. Red blood cells were lysed using 1.5 mL of ACK buffer per well. Single-cell suspensions were then stained for cell-surface markers using antibodies listed below. Cells were stained in 50μL FACS buffer with 1:250 antibody concentration per sample, FACS buffer to be run by flow cytometry (BD FACSAria)

### Flow cytometry

Individual cells were sorted initially using FSC/SSC gating and live cells sorted via live-dead stain exclusion (Propidium iodide). Cell cytometric data was analyzed using the FlowJo software platform. For the purposes of this experiment, resident cardiac macrophages were identified with the following profile: CD45^+^CD64^+^CD11b^+^CCR2^-^MHCII^Lo^TIMD4^+^LYVE1^+^. A completed FCSminiINFO checklist for the flow cytometry experiments is provided as **Supplementary Material 1**.

### Mouse antibodies used

CD64 - Brilliant Violet 421 anti-mouse (FcγRI)

MHCII - MHC Class II (I-A/I-E) Monoclonal Antibody (M5/114.15.2), Super Bright 600, eBioscience™

CD45 - FITC anti-mouse CD45.2 (Clone 104)

CCR2 - BD OptiBuild BB700 Rat Anti-Mouse CD192

LYVE1 - LYVE1 Monoclonal Antibody (ALY7), PE-Cyanine7, eBioscience™

TIMD4 - PE anti-mouse Tim-4 (RMT4-54)

CD11b - APC/Cyanine7 anti-mouse/human CD11b (M1/70)

### Nanostring analysis

Resident cardiac macrophages intended for NanoString analysis were isolated from cell digests of 3 hearts per time point (control, 2 days post AMI, and 4 days post-AMI). The pooled samples were separated using the FACS gating strategy described above. From these cells, 100 ng of RNA per sample was extracted and NanoString analysis was performed by the University of Hawai’i Cancer Center Genomics and Bioinformatics Shared Resource (GBSR) core facility with the NanoString nCounter. Multiplex transcriptional analysis of inflammatory genes was carried out by employing the nCounter Myeloid Innate Immunity Panel which consists of 754 different mouse genes that correspond with the innate immune response (**Supplementary Figure 1**, **Supplementary Material 2: Table 1**). More information describing the specifics of NanoString technology can be found in their website. Transcriptional data was initially evaluated with nSolver software and further analyzed using ROSALIND. The NanoString data generated in this study have been deposited in the Gene Expression Omnibus under accession number GSE333790.

### VISIUM spatial gene expression analysis

Perfused whole heart samples collected from control mice and after 2 days and 4 days of AMI were embedded in OCT (TissueTek), and cryosectioned into 10 μm slices at −10 °C. Sections were mounted on a chilled Visium Spatial Gene Expression slide (10x Genomics) adhesion of cryosections occurred as the slide warmed. Tissue sections were then fixed in chilled methanol and stained according to the manufacturer’s specifications. An optimal tissue permeabilization time of 18 minutes was determined experimentally and all samples were permeabilized according to the Visium Tissue Optimization manufacturer protocols. Tissue optimization experiments entailed validation of sample integrity using images captured using a TRITC filter in tandem with fluorescence microscopy (Zeiss AxioSkop 2 Plus). Brightfield histology images were also taken after H&E staining as spatial reference by 10x Genomics. cDNA libraries were generated using Visium Spatial Gene Expression slide & Reagent Kit according to the manufacturer’s instructions (10x Genomics) and sequenced on a NextSeq 500 system (Illumina). This resulted in an average read depth of 71,274 reads/spot (control), 66,264 reads/spot (2 days post-AMI), and 82,181 reads/spot (4 days post-AMI). Spatial transcriptomic data was initially analyzed with Space Ranger and further using the BioTuring Lens suite of bioinformatic tools. Normalization of batch effects between samples was achieved using the integrated Harmony algorithm. Macrophage-associated spatial signatures were assigned using combinations of established marker genes associated with the macrophage populations analyzed here. Because each Visium spot may contain transcripts from multiple cells, these assignments were interpreted as spatial transcriptional signatures rather than definitive single-cell macrophage identities.

### Statistical analysis

Because RCM samples were pooled before NanoString profiling, NanoString differential-expression results were interpreted as an exploratory transcriptional screen. ROSALIND was used for normalization, fold-change estimation, adjusted P-value calculation, and pathway-enrichment analysis to identify candidate transcriptional changes between time points. Background correction is achieved by computing the standard deviation and mean of built-in negative controls. Positive control normalization relies on synthetic “spike-in” control proteins adjusts for sample variation. In this case, a lane-specific normalization factor is derived from the geometric mean of positive controls. Codeset (housekeeping) normalization employs the geNorm algorithm to identify the most stable subset of housekeeping genes for each sample (22). Lane-specific factor application multiplies the raw counts by sample specific normalization factors which are calculated by dividing the global geometric mean of normalizer probes by sample-specific geometric mean. Lastly, covariate correction minimizes batch effects by isolating the gene of interest while correcting for unwanted background factors and batch effects across pools. Adjusted P-value (P-adj) or differential gene expression is calculated using Benjamini-Hochberg method of estimating false discovery rates (FDR) which limits detection of false positives in large samples. The ROSALIND platform employs gene-set analysis (GSA) as well as FDR and p-Elim pruning to filter out background noise and local gene ontology dependencies. Therefore, only significantly enriched pathways are returned despite a small number of input genes. All non-transcriptomic data are represented as Mean ± SEM. Statistical analysis was performed using GraphPad Prism 8 software. To determine significance, unpaired, two-tailed Welch’s t-test was performed when comparing experimental and control groups. *P<0.05, **P<0.005, ***P<0.0005, ****P<0.0001.

## Results

### Fluctuations in the number of RCM correlate with different stages of AMI

We employed an ischemia/reperfusion (IR) model to study RCM physiology, which relied on temporary occlusion of the LAD. Cardiac tissues were reperfused following 30 minutes of LAD ligation, and the cells were liberated by enzymatic digestion. We believe this infarction model best recapitulates the clinical manifestations of AMI followed by therapeutic reperfusion. A novel subpopulation of RCM was recently identified and is characterized by the following cell surface marker expression profile: CD45^+^CD64^+^CD11b^+^CCR2^-^MHCII^Lo^TIMD4^+^LYVE1^+^ (15). These macrophages are purported to play a critical part in orchestrating complex cell and tissue responses in AMI and IRI (4, 15). As such, we utilized the aforementioned cell surface markers to isolate RCM using FACS from the non-infarcted heart and after 0, 2-, and 4-, days post-AMI (**Figure 1A**). We found that the absolute number of this specific macrophage phenotype was significantly decreased from control to 4-day and also from 2- and 4-day post-AMI samples (**Figure 1B**). While these cells have been shown to decrease in numbers after two days of infarction, a seminal paper by Dick et al. demonstrated that RCM identified with the same markers undergo a proliferative burst during this timeframe as well (15).

**Figure 1:**
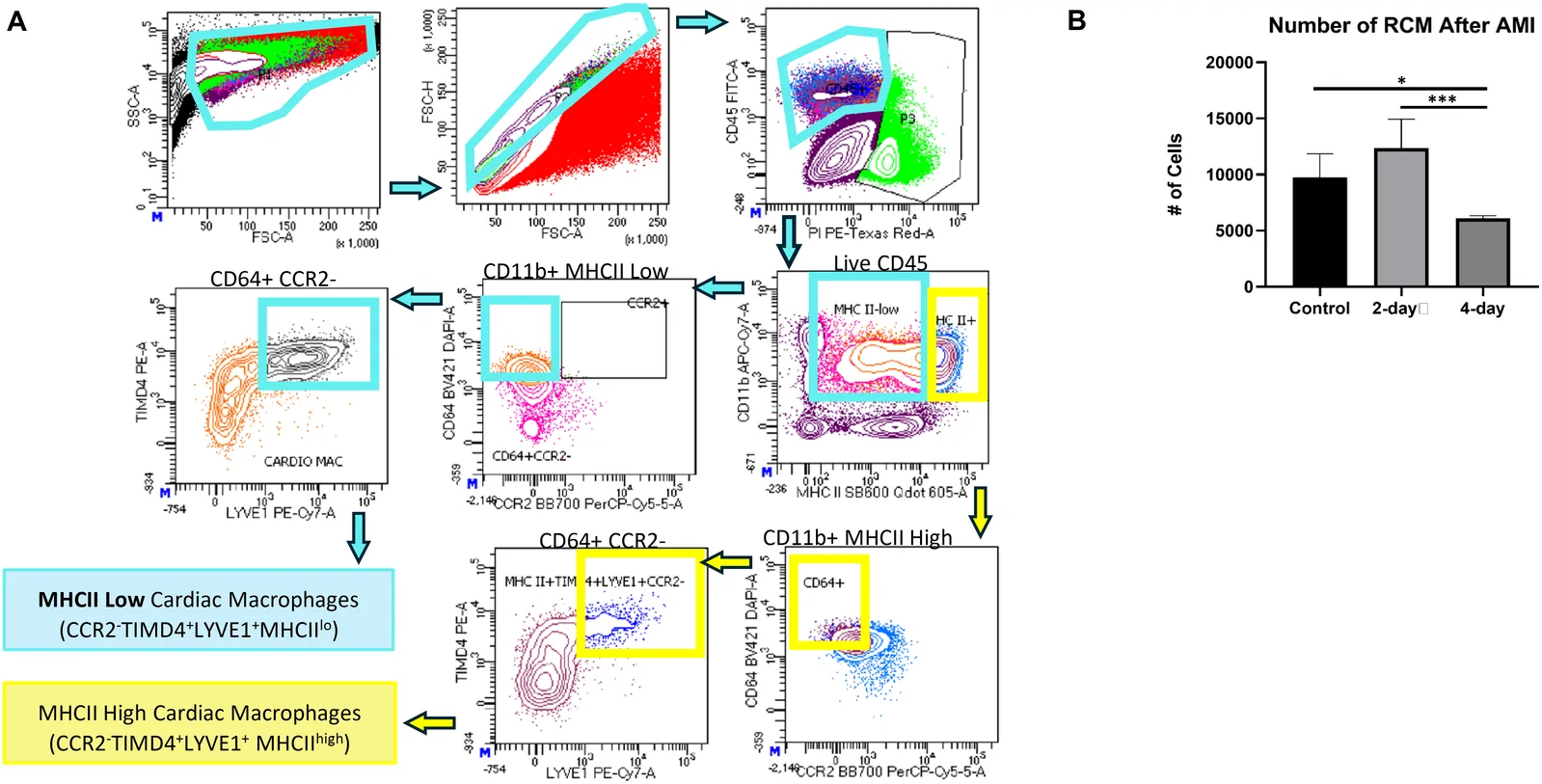
Isolation of resident cardiac macrophages using flow cytometry. **(A)** After enzymatic digestion of infarcted mouse hearts, cells were sorted using the FACSAria flow cytometer. The gating strategy was designed to specifically select for macrophages with a specific signature of surface-markers: CD45^+^CD64^+^CD11b^+^CCR2^-^MHCII^Lo^TIMD4^+^LYVE1^+^. This population of macrophages is indicative of RCM used for NanoString analysis. Image provided courtesy of 10x Genomics, Inc. **(B)** Absolute number of RCM collected from control and infarcted mouse hearts after 2 and 4 days of reperfusion following MI. RCM number was significantly lower after 4 days when compared to control and 2 day samples. *p < 0.05, ***p < 0.005, n=3 for all time points and control.

### Multiplex mRNA analysis of RCM reveals transcriptional changes corresponding with the phasic responses of macrophages during AMI

In general, macrophages comprise only a small percentage of the total cells in the steady-state heart; even after a large-scale macrophage recruitment to the infarcted heart, the percentage remains relatively small (23, 24). Nevertheless, given the important role of RCM in the steady-state heart and in disease conditions, we sought to determine relevant transcriptional changes associated with AMI. To achieve this, NanoString mRNA analysis was carried out with the nCounter^®^ Mouse Myeloid Innate Immunity Panel V2 which probed for 754 genes (plus 20 control genes) related to myeloid cell physiology (**Supplementary Figure 1A**). FACS-sorted RCM were recovered from three mouse hearts per time point and pooled before RNA extraction to obtain sufficient RNA for NanoString profiling. Therefore, NanoString results were interpreted as an exploratory transcriptional screen rather than as an animal-level replicated differential-expression experiment. We then used the ROSALIND software, which allowed for differential analysis of genes between time points to analyze the NanoString transcriptional data (**Figure 2**). A fold-change cutoff of ±2 with an adjusted P-value threshold of 0.05 was used to identify genes meeting exploratory differential-expression thresholds for each time point. We identified 30 genes that met the exploratory fold-change and adjusted P-value thresholds when comparing control and 2-day samples (**Figure 2A**).

**Figure 2:**
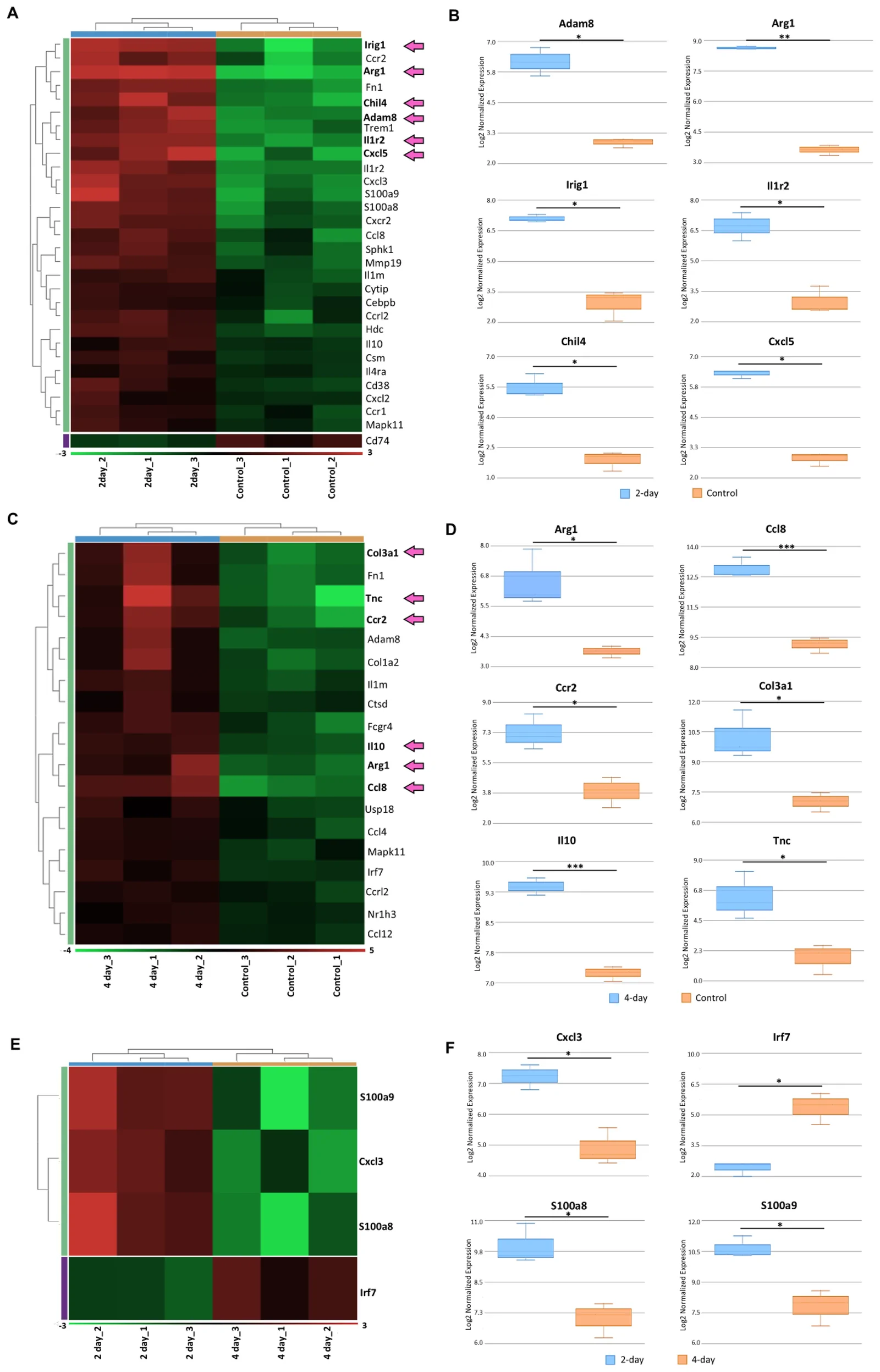
Multiplex mRNA analysis of RCM reveals transcriptional changes corresponding with the phasic responses of macrophages during early-onset AMI. **(A, B)** Heat map and top differentially expressed genes (DEG) between RCM collected from control samples compared to samples after 2 days of AMI. **(C, D)** Heatmap, and top DEG comparing RCM collected from control samples and compared to samples after 4 days of AMI. **(E, F)** Heat map, and top DEG comparing RCM collected after 2 and 4 days of AMI. Statistical significance was determined using an FDR cutoff threshold of 0.05 and graphical values depict Log2 of normalized gene expression in comparative samples., n=3 pooled samples of 3 hearts for all time points and control. *P-adj < 0.05, **P-adj < 0.01, ***P-adj < 0.005.

While 29 of 30 genes upregulated were largely related to transcription of pro-inflammatory cytokines such as Irg1 (FC: 38.36, p-adj 0.02), Il1r2 (FC: 23.00, p-adj 0.02), and Adam8 (FC: 19.18, p-adj 0.03), upregulation in anti-inflammatory genes was also observed (**Figure 2B**). When comparing control with 2-day samples of RCM, the largest statistically significant increases were seen in Arg1 (FC: 43.44, p-adj 8.0×10^—3^), Chil4 (FC: 42.00, p-adj 0.05) and Cxcl5 (FC: 22.10, p-adj 0.05). While IL10 transcription was also significantly upregulated, albeit to a lesser extent (FC: 3.84, p-adj 4.6x10^3^). The macrophage marker CD74 was the only significantly downregulated gene 2 days after AMI (FC: -2.61, p-adj 0.02).

Differential expression analysis of RCM samples collected 4 days after AMI compared to control samples revealed 19 genes that were significantly upregulated (**Figure 2C**). The anti-inflammatory genes, Arg1 (FC: 12.44, p-adj 0.03) and IL10 (FC: 4.54, p-adj 1.90×10^-3^) were significantly upregulated in 4-day samples (**Figure 2D**). Ccr2, which is typical of infiltrating macrophages, was also significantly upregulated (FC: 14.00, p-adj 0.03).

Not surprisingly, transcription of genes involved in regulating cardiac tissue repair like Tnc (FC: 89.00, p-adj 0.03), CCL8 (FC: 14.07, p-adj 1.90×10^-3^), Fn1 (FC: 11.67, p-adj 0.03), and Col3a1 (FC: 11.53, p-adj 0.03) were all significantly upregulated (**Figure 2D**). Lastly, transcriptional analysis comparing differences between RCM isolated after 2 days and 4 days of infarction revealed significantly elevated transcription of only 4 genes (Figre 2E). Transcription of S100a8 (FC: 8.18, p-adj 0.05), S100a9 (FC: 6.76, p-adj 0.05), and CXCL3 (FC: 5.52, p-adj 0.04) were all downregulated in RCM after 4 days of AMI compared to 2 days, while Irf7 transcription was significantly upregulated in the 4-day samples (FC: 2.91, p-adj 0.04) when compared to RCM 2 days post-AMI (**Figure 2F**).

### Pathway enrichment and transcription factor activity analysis of RCM

The Rosalind software platform employs a number of publicly available datasets which allows for analysis of pathway enrichment associated with differentially expressed genes. WikiPathways and Reactome are 2 of the most widely used databases for inferring cell functions and regulatory pathways. We elected to use both data sets in our analyses because they provided us with the most statistically significant pathway hits with each having its unique strengths in leveraging multiple resources to offer a more comprehensive view of biological activity.

The 30 statistically significant genes in RCM samples after 2 days of AMI compared with control samples were previously determined using differential expression analysis, and further characterized in terms of pathway enrichment (**Table 1**). According to WikiPathways, the top 3 enriched pathways when comparing RCM after 2 days of AMI to control samples are: 1) “Amino Acid Metabolism”, which involved Arg1 and Hdc; 2) “Circulating monocytes and cardiac macrophages in diastolic dysfunction”, regulated by CCR2 and IL10; and 3) “GPCRs, Class A Rhodopsin-like” involving CCR2, CXCR2, CCL2 and CCR1 (**Table 1A**). Analysis of the samples using the Reactome database suggested the top 3 enriched pathways to be: 1) “Metal sequestration by antimicrobial proteins” through transcription of S100a8 and S100a9; 2) “RHO GTPases Activate NADPH Oxidases” related to upregulation of S100a8, S100a9, and Mapk1; and 3) “Chemokine receptors bind chemokines” associated with upregulation of CXCL3, CXCL5, CXCR2, CXCL2, AND CCRL2 (**Table 1A**). CD74 was the only statistically significant gene to be down regulated in 2-day RCM samples compared with controls. Whereas pathway enrichment with WikiPathways showed this gene to be involved in the “Macrophage markers” pathway, the same analysis with the REACTOME database assigned CD74 to pathways named “Cell surface interactions at the vascular wall” and “MHC class II antigen presentation” (**Table 1A**).

**Table 1:** Pathway analysis of NanoString results from RCM samples during early-onset AMI.

A		
2 Day vs Control		
Dataset	Pathway	Gene
WikiPathways	Amino Acid metabolism	Arg1, Hdc
	Circulating monocytes and cardiac macrophages in diastolic dysfunction	Ccr2, Il-10
	GPCRs, Class A Rhodopsin-like	Ccr2, Cxcr2, Ccl2, Ccr1
REACTOME	Metal sequestration by antimicrobial proteins	S100a8, S100a9
	RHO GTPases Activate NADPH Oxidases	S100a8, S100a9, Mapk1
	Chemokine receptors bind chemokines	Cxcl3, Cxcl5, Cxcr2, Cxcl2, Ccrl2

B		
4 Day vs Control		
Dataset	Pathway	Gene
WikiPathways	Circulating monocytes and cardiac macrophages in diastolic dysfunction	Ccr2, Il-10
	Inflammatory Response Pathway	Fn1, Col3a1, Col1a2
	Dysregulated miRNA Targeting in Insulin/PI3K-AKT Signaling	Col3a1 Col1a2
REACTOME	ECM proteoglycans	Tnc, Fn1, Col3a1, Col1a2
	Extracellular matrix organization	Fn1, Col3a1, Col1a2
	MET activates PTK2 signaling	Fn1, Col3a1, Col1a2

C		
2 Day vs 4 Day		
Dataset	Pathway	Gene
WikiPathways	Cytokines and Inflammatory Response	Cxcl3
	Apoptosis	Irf7
REACTOME	Metal sequestration by antimicrobial proteins	S100a8, S100a9
	RHO GTPases Activate NADPH Oxidases	S100a8, S100a9
	Regulation of TLR by endogenous ligand	S100a8, S100a9

A-C) Using the on-board pathway enrichment tools and data sets, we analyzed the genes returned from differential expression comparisons made between time points. Statistical significance of pathway enrichment was set at p ≤ 0.05 and only genes meeting that statistical threshold were included.

Pathway enrichment analysis of the 19 statistically significant genes in RCM samples collected after 4 days of AMI compared to control samples using WikiPathways showed; 1) “Circulating monocytes and cardiac macrophages in diastolic dysfunction” determined by transcription of CCR2 and IL10; 2) “Inflammatory Response Pathway” involving transcription of Fn1, Col3a1, and Col1a2; and 3) “Dysregulated miRNA Targeting in Insulin/PI3K-AKT Signaling” associated with upregulated transcription of Col3a1 and Col1a2 (**Table 1B**). REACTOME analysis of these samples showed that the most enriched pathways were: 1) “ECM proteoglycans” through Tnc, Fn1, Col3a1, Col1a2; 2) “Extracellular matrix organization” related to transcription of Fn1, Col3a1, Col1a2; and 3) “MET activates PTK2 signaling” transcription of the same genes (**Table 1B**).

Differential analysis of RCM between 2-day and 4-day samples post-AMI revealed 4 statistically significant genes. Using the WikiPathways database we found: 1) “Cytokines and Inflammatory Response” to be enriched, and related to the down regulation of CXCL3 in 4-day samples; and 2) “Apoptosis” pathway was also enriched via upregulation of Irf7 in RCM after 4 days of AMI compared to samples after 2 days (**Table 1C**). Analysis of statistically significant genes from differential analysis of these time points with REACTOME demonstrated that: 1) “Metal sequestration by antimicrobial proteins”; 2) “RHO GTPases Activate NADPH Oxidases”; and 3) “Regulation of TLR by endogenous ligand” were the most highly enriched pathways due in large part to significantly higher expression of S100a8 and S100a9 in 2-day samples compared to 4-day samples (**Table 1C**).

### Spatiotemporal analysis demonstrates distinct transcriptional expression patterns of macrophages during AMI

The Visium platform by 10X Genomics enabled us to examine gene transcription levels with spatial context over time. To achieve this, we prepared 10 µm thick cryosections of cardiac tissue from control mice in the steady state and experimental animals after 2, and 4 days following 30 minutes of LAD ligation to simulate a clinical representation of acute ischemia and reperfusion (**Figure 3A**). BioTuring Lens is a powerful software that leverages sophisticated computational algorithms and powerful cloud computing to analyze spatial transcriptomic data. The Harmony algorithm is implemented within BioTuring Lens to minimize batch effect and facilitate integration of multiple samples across conditions or time points, as in the case with our analysis. Thus, we are able to accurately perform a spatiotemporal comparison of samples to demonstrate transcriptional changes over time with spatial context in the heart during critical early stages of AMI. From each cryosection, the Visium platform captured 2131 spots under tissue in the steady state sample, 1880 spots after 2 days of AMI and 2258 spots after 4 days (**Figure 3B**). This resulted in an average read depth of 71,274 reads/spot, 66,264 reads/spot, and 82,181 reads/spot in the steady state, after 2 days, and 4 days post-AMI respectively (**Figure 3C**). The Visium analysis identified a median of 1584 genes/spot obtained from control samples, 2214 genes/spot from samples taken 2 days post-AMI, and 2738 genes/spot 4 days post-AMI (**Figure 3D**). Masson’s trichrome staining of tissue samples at different time points reveals the degree location of cardiac tissue injury (**Figures 3E–G**). We then analyzed the gene expression patterns of 22 well-established, macrophage-associated marker genes to analyze the changing localization patterns of macrophages and their progenitors within the infarcted heart (**Supplementary Figure 1B**). This provided us with an aggregate view of the spatiotemporal distribution of macrophage-associated genes. We observed that in the steady state, both macrophages and monocytes are rather ubiquitous and are not characterized by any particular concentration pattern (**Figure 3H**). In the AMI sample after 2 days, macrophages began to accumulate in the infarct zone, and by 4 days were highly abundant and concentrated in the infarct zone radiating outward indicating larger scale tissue inflammation beyond the actual injury site (**Figures 3I, J**).

**Figure 3:**
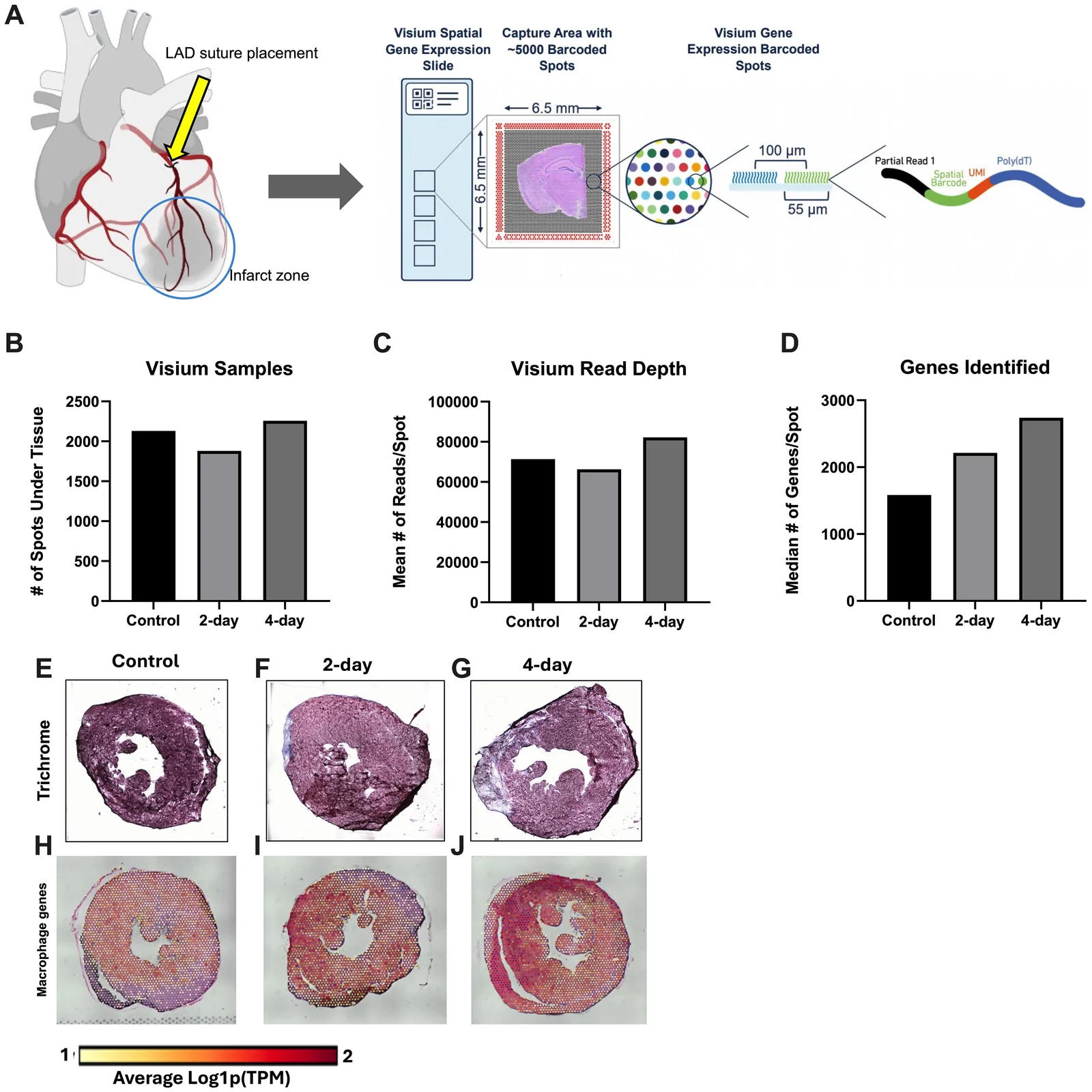
Spatiotemporal analysis demonstrates distinct transcriptional expression patterns of macrophages during early-onset AMI. **(A)** Schematic depicting LAD ligation surgery and sample preparation for Visium analysis. **(B)** Graphical representation illustrating the percentage of spots covered by tissue. **(C)** Quantification of read depth per sample represented as mean number of reads per spot achieved by the bioanalyzer. **(D)** The median number of genes captured by each spot. **(E–G)** Masson’s trichrome staining of tissue samples. **(H–J)** Spatiotemporal distribution of 22 different macrophage-related genes. Gene expression is represented the average Log1p (TPM).

### Macrophage heterogeneity and distribution of subtypes during AMI

Further analysis of macrophage subtypes was carried out using cell-surface marker genes which are indicative of 6 major macrophage phenotypes characterized by their functionality (**Supplementary Figure 1C**). Mean expression of macrophage-specific genes was used to identify the spatiotemporal expression patterns of RCM, antigen presenting macrophages, infiltrating macrophages, pro-inflammatory “M1” macrophages, anti-inflammatory “M2” macrophages, and interferon-related (IFN) macrophage populations during early stages of AMI. Transcription of RCM-associated genes such as Timd4, Lyve1, Folr2, Cbr2, and F13a1 was highest in the steady state, and then decreased over time (**Figures 4A, B**). In the steady state sample, RCM markers appeared to be randomly distributed. However, after 2 days RCM gene transcription patterns began to be focused near the infarcted area, and by 4 days were highly concentrated in the infarct zone. Expression of RCM-associated gene decreased as the AMI immune response entered the inflammatory and proliferative stages (**Figure 4C**).

**Figure 4:**
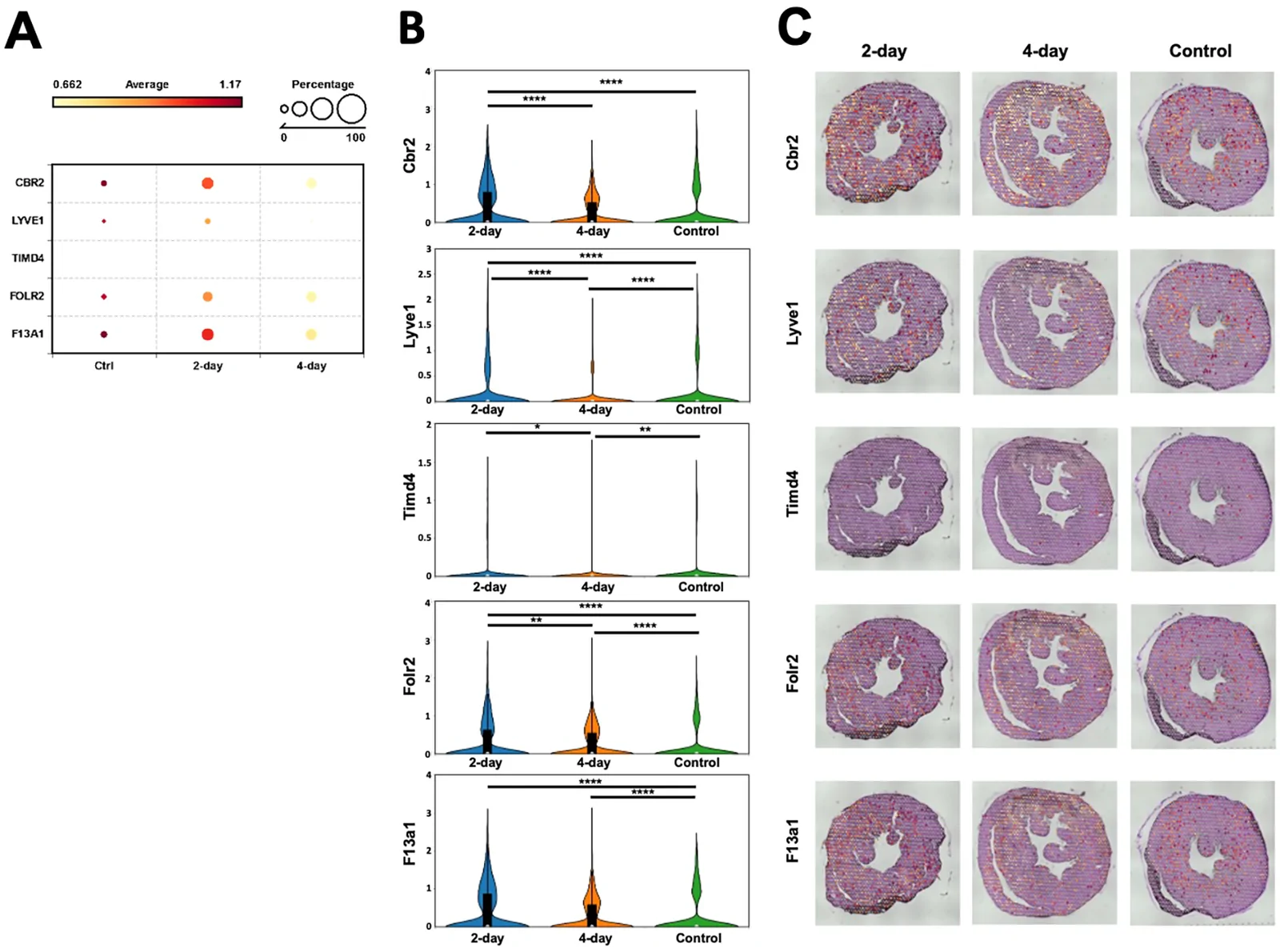
Spatiotemporal abundance and distribution of RCM markers during early-onset AMI. **(A)** Bubble heatmap of RCM specific genes depicting expression level as the average -LogTPM (transcripts per million) darker color represents higher expression. Bubble size indicates percentage of cells expressing each gene (coverage of gene distribution throughout the tissue samples. **(B)** Violin plots of RCM gene expression at each time point, error bars represent 95% confidence intervals. **(C)** Spatial distribution and average expression of RCM genes during early-onset AMI color intensity reflects average gene expression. *P< 0.05, **P< 0.01, ***P< 0.001, ****P< 0.0001.

Expression of gene markers associated with antigen presentation including H2-Eb1, H2-Aa, H2-Ab1, and Cd74, was reduced in the 2-day samples compared with steady state heart, and then increased again in 4-day samples (**Figures 5A, B**). Transcription of each gene had a random spatial signature in control and 2-day samples, but, after 4 days was clearly localized in the infarct zone (**Figure 5C**).

**Figure 5:**
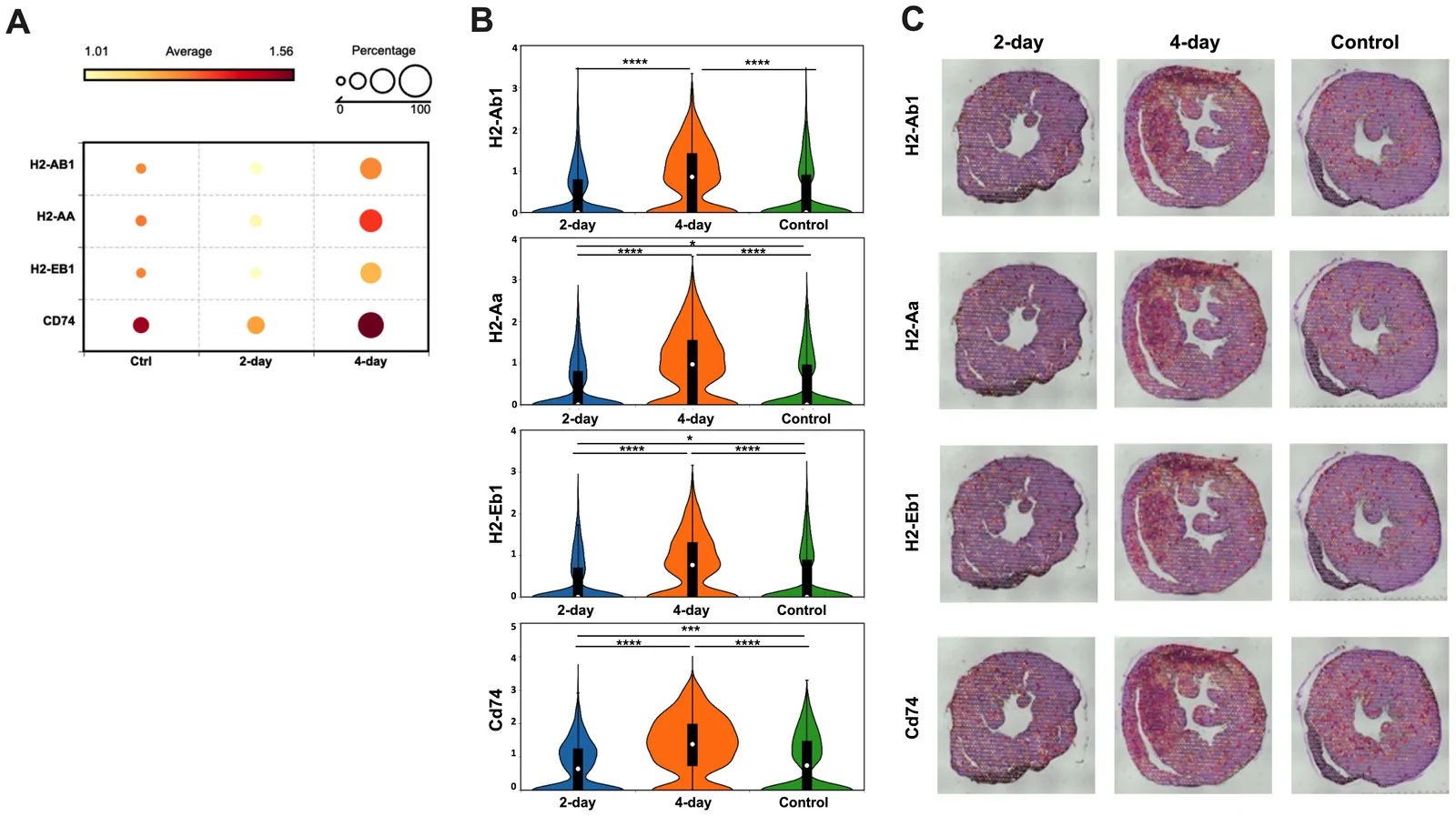
Antigen-presenting macrophage marker expression Is enhanced and heavily localized within the infarct zone. **(A)** Bubble heatmap of APM specific genes depicting expression level as the average -LogTPM (transcripts per million) darker color represents higher expression. Bubble size indicates percentage of cells expressing each gene (coverage of gene distribution) throughout the tissue samples. **(B)** Violin plots of APM gene expression at each time point, error bars represent 95% confidence intervals. **(C)** Spatial distribution and average expression of RCM genes during early-onset AMI color intensity reflects average gene expression. *P< 0.05, ***P< 0.001, ****P< 0.0001.

Interestingly, gene expression of Ccr2, Cx3cr1, and Ptprc (Cd45) which are indicative of infiltrating monocyte-derived macrophages remained largely unchanged over time although the number of cells expressing these genes expanded (**Figures 6A, B**). Spatial distribution of all 3 genes showed sparse expression in both control and 2-day samples, but, by 4 days were concentrated in the injury site (**Figure 6C**).

**Figure 6:**
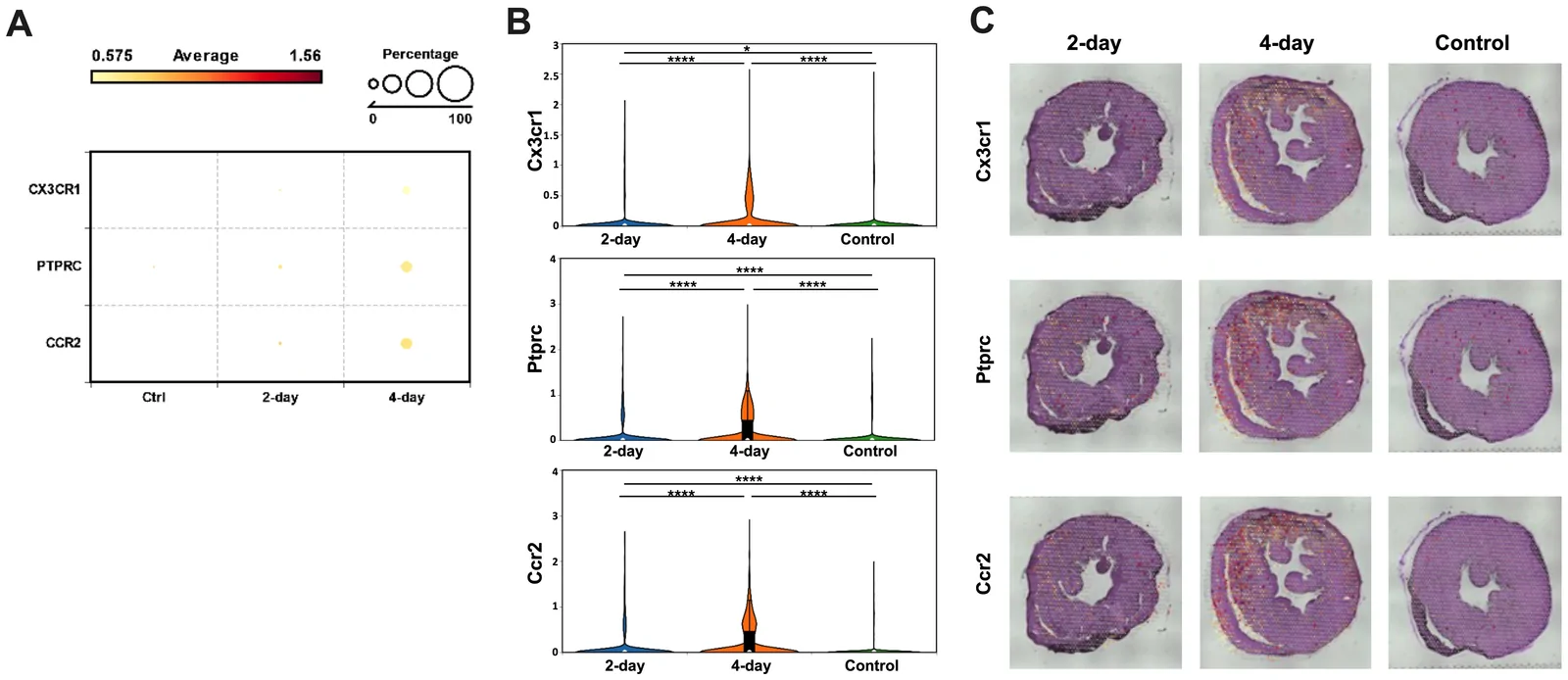
Minimal expression and distribution of infiltrating macrophage markers. **(A)** Bubble heatmap of IM specific genes depicting expression level as the average -LogTPM (transcripts per million) darker color represents higher expression. Bubble size indicates percentage of cells expressing each gene (coverage of gene distribution) throughout the tissue samples. **(B)** Violin plots of IM gene expression at each time point, error bars represent 95% confidence intervals. **(C)** Spatial distribution and average expression of IM genes during early-onset AMI color intensity reflects average gene expression. *P< 0.05, ****P< 0.0001.

We also observed that “classical” or “M1” pro-inflammatory macrophage markers like Cd68 and Adgre1 (F4/80), exhibited very little expression in the control sample, although transcription increased over time. Other pro-inflammatory macrophage genes like Lamp2 (Mac3), had an elevated baseline expression in the steady state compared to the other 2 time points (**Figure 7A**). While elevated expression of both genes was evident in 2- and 4-day time points, mean expression values never returned to control transcription values (**Figure 7B**). Spatial analysis indicated that, while Cd68 and Adgre1 were sparsely and randomly distributed in the steady state sample, gene expression eventually migrated toward the infarct area after 2 days, and by 4 days both genes were highly concentrated in the infarct zone, especially Cd68 (**Figure 7C**). Lamp2, on the other hand, exhibited the highest expression in control sample, and also had similar spatial signatures. Genes were distributed randomly and ubiquitously in control and 2-day samples, but in 4-day samples both Cd68 and Lamp2 were strongly expressed in the infarct zone as well (**Figure 6C**).

**Figure 7:**
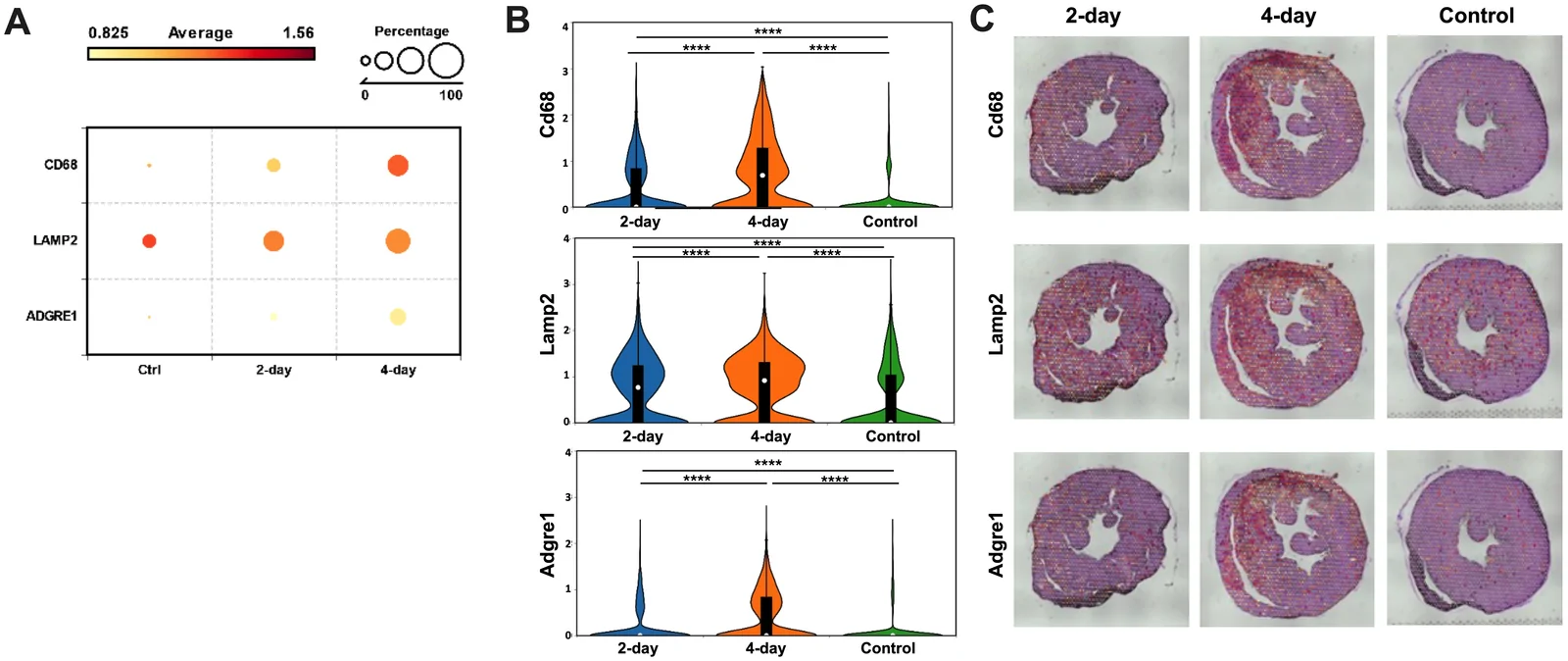
Moderate increase in expression of M1 macrophage markers correlates with infarct progression. **(A)** Bubble heatmap of APM specific genes depicting expression level as the average -LogTPM (transcripts per million) darker color represents higher expression. Bubble size indicates percentage of cells expressing each gene (coverage of gene distribution) throughout the tissue samples. **(B)** Violin plots of M1 gene expression at each time point, error bars represent 95% confidence intervals. **(C)** Spatial distribution and average expression of M1 genes during early-onset AMI color intensity reflects average gene expression. ****P< 0.0001.

While the expression of Fabp5, Timp2, Gpnmb, and Trem2 were associated with M2 macrophage polarization, no expression of the latter 2 genes was seen in the steady state sample with increasing expression in each subsequent time point (**Figure 8A**). Fabp5 and Timp2, on the other hand, showed elevated transcription levels even in the control and 2-day samples with expression of both genes being highest at day 4 (**Figures 8A, B**). Spatial gene expression patterns for all genes except for Timp2 colocalized with the infarct zone over time. Timp2 showed random distribution in both the steady-state and 2-day samples, but by 4 days post-AMI expression was most intense in the infarct zone (**Figure 8C**). These results suggest that populations of M2 macrophages both originate in and infiltrate the cardiac compartment, more specifically the infarct zone during AMI.

**Figure 8:**
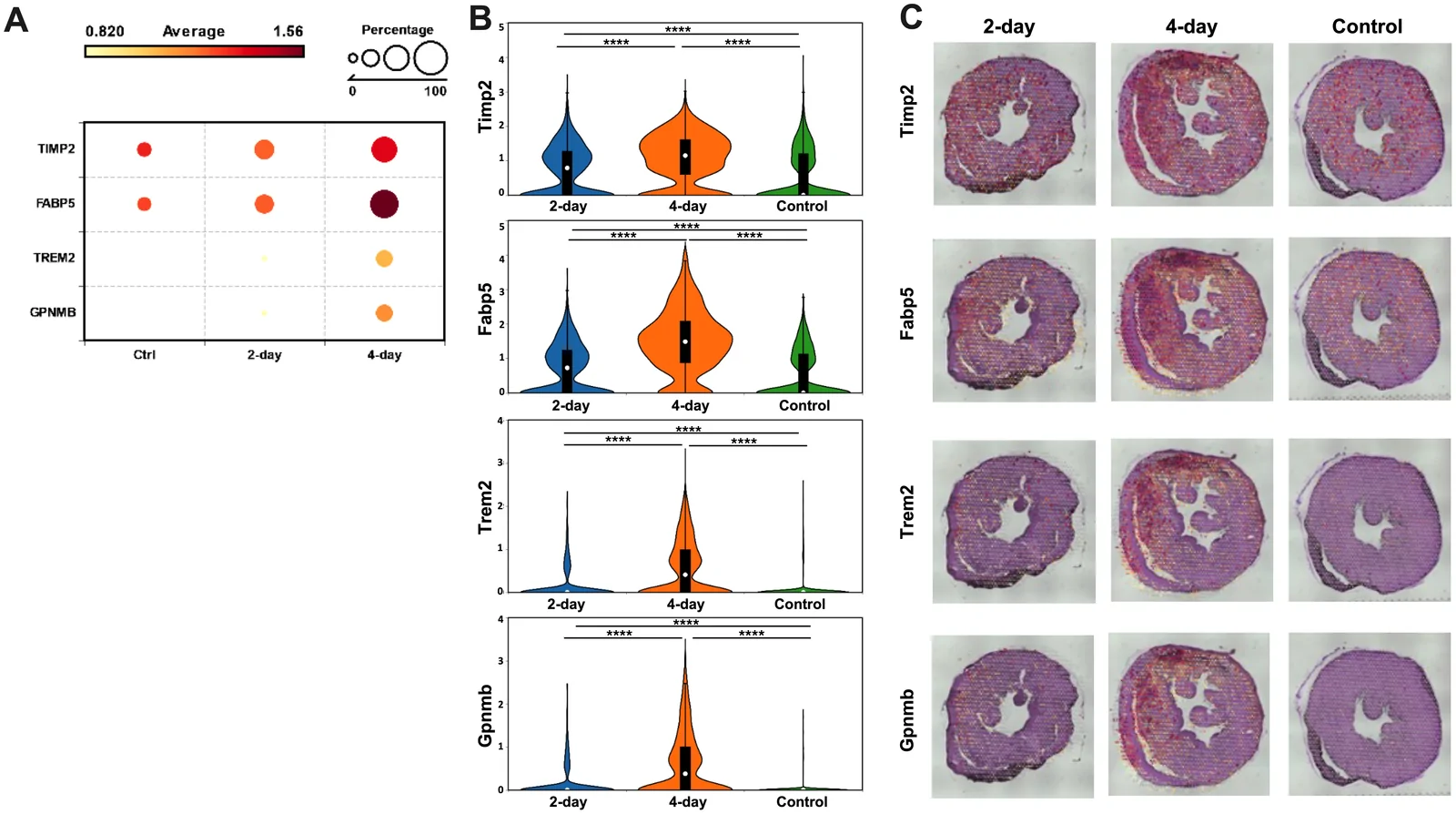
M2 macrophage genes are recruited to the infarct zone at the earliest stages of AMI. **(A)** Bubble heatmap of M2 specific genes depicting expression level as the average -LogTPM (transcripts per million) darker color represents higher expression. Bubble size indicates percentage of cells expressing each gene (coverage of gene distribution) throughout the tissue samples. **(B)** Violin plots of M2 gene expression at each time point, error bars represent 95% confidence intervals. **(C)** Spatial distribution and average expression of M2 genes during early-onset AMI color intensity reflects average gene expression. ****P< 0.0001.

Interferon-related genes, Irf7, Isg15, and Ifit2, displayed minimal expression in the steady state with a slight increase in 2-day samples, but exhibited high expression levels after 4 days of AMI (**Figures 9A, B**). Spatial gene analysis showed a random distribution of all 3 genes in both control and 2-day samples. After 4 days post-AMI, transcription of IFN genes was concentrated in specific parts of the infarcted area. More specifically, the expression of IFN genes in our 4-day sample appeared highest in what is commonly referred to as the peri-infarct zone (**Figure 9C**), which suggests that IFN-associated macrophages are important during the transition from inflammation to repair during the early stages of AMI.

**Figure 9:**
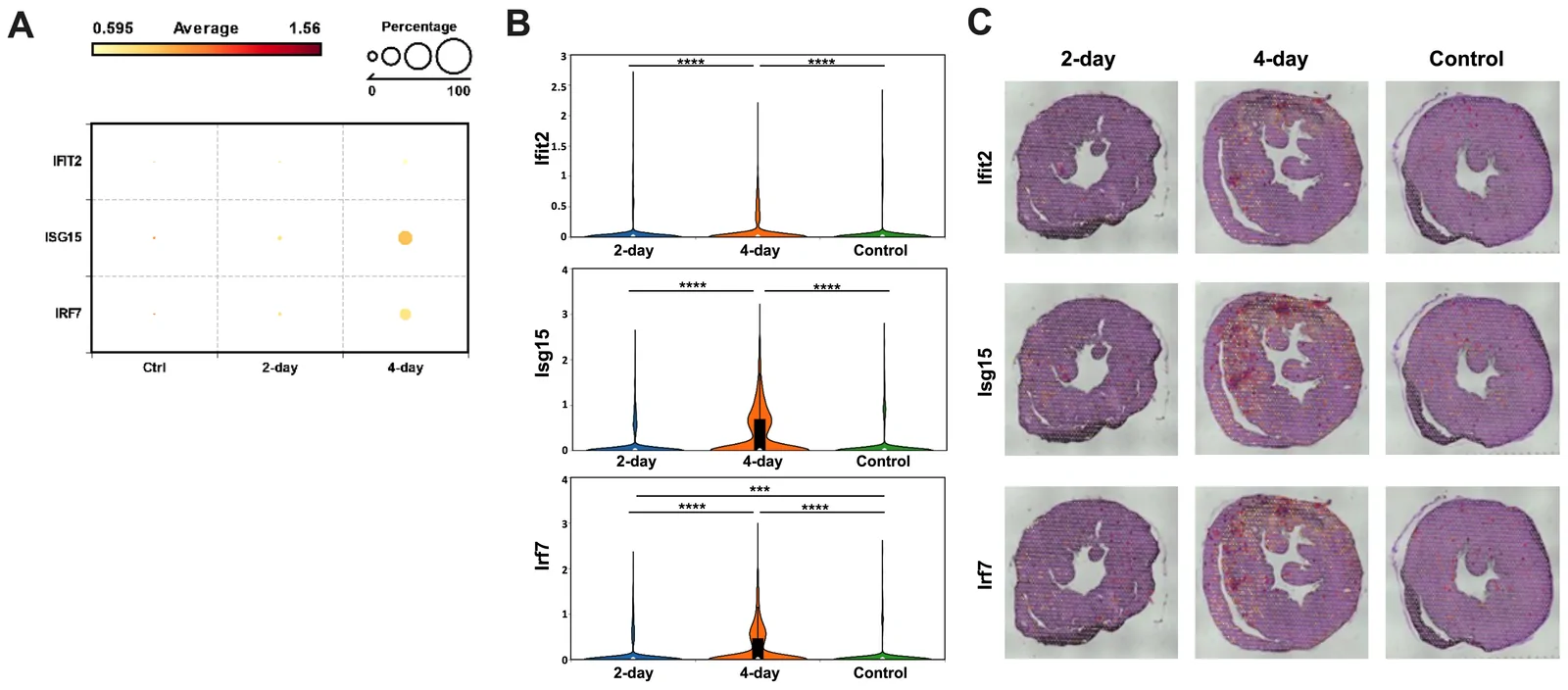
Interferon associated macrophages or minimally expressed throughout early-onset AMI. **(A)** Bubble heatmap of interferon **(IFN)** specific genes depicting expression level as the average -LogTPM (transcripts per million) darker color represents higher expression. Bubble size indicates percentage of cells expressing each gene (coverage of gene distribution) throughout the tissue samples. **(B)** Violin plots of IFN gene expression at each time point, error bars represent 95% confidence intervals. **(C)** Spatial distribution and average expression of IFN genes during early-onset AMI color intensity reflects average gene expression. ***P< 0.001, ****P< 0.0001.

### Localization of S100 transcripts and macrophage profile

Results from our multiplex transcriptional analysis (NanoString) of RCM revealed that S100a8 and S100a9 are among the most upregulated genes expressed during AMI. Current studies have demonstrated disparate roles for these proteins in both the progression and resolution of myocardial damage following an AMI event (25–27). Interestingly, while differential expression analysis revealed statistically significant upregulation of S100a8 and S100a9 in RCM samples after 2 days of AMI compared to samples taken after 4 days, the opposite was the case with our Visium analysis. It is known that S100a8 is highly labile compared to S100a9, therefore the latter was used to observe expression levels and spatial dynamics over time. In the Visium samples, comparative analysis within BioTuring Lens showed strong spatial co-localization of S100a9 with macrophage-associated markers, including Csf1r, Cd68, and Adgre1, particularly after 2 and 4 days of IRI (**Figure 10A**). Because individual Visium spots may contain transcripts from multiple cell types, these findings were interpreted as a macrophage-associated S100a9 spatial signature rather than definitive evidence that S100a9 expression originated from resident cardiac macrophages. We found that spatiotemporal distribution of S100a9 expression colocalized with tissue injury, and was expressed by fewer cells but at higher levels in control samples (**Figures 10B, C**). Over time, the number of macrophages expressing S100a9 increased although gene expression itself declined (**Figure 10C**). Using IHC, we were able to visualize S100a9 expression in murine heart sections after 0 (control), 2, and 4 days. This was compared with localization of pan-macrophage marker, CD68, which we found correlated with our transcriptional results. These findings provide orthogonal protein-level evidence of spatial association between S100A9 and CD68-positive macrophage-rich regions, but do not distinguish resident cardiac macrophages from infiltrating monocyte-derived macrophages. IHC visualization revealed a highly localized pattern corresponding with infarcted tissue (**Figure 10D**). Quantification of IHC samples was carried out using ImageJ software and measuring pixel-area coverage (px^2^). Our analyses demonstrated protein expression levels of S100a9 that corresponded to transcription patterns over time (**Figure 10E**). Delayed tissue expression of S100a8, S100a9, and Irf7 in cardiac tissues was temporally associated with macrophage-associated transcriptional responses during the early phases of AMI.

**Figure 10:**
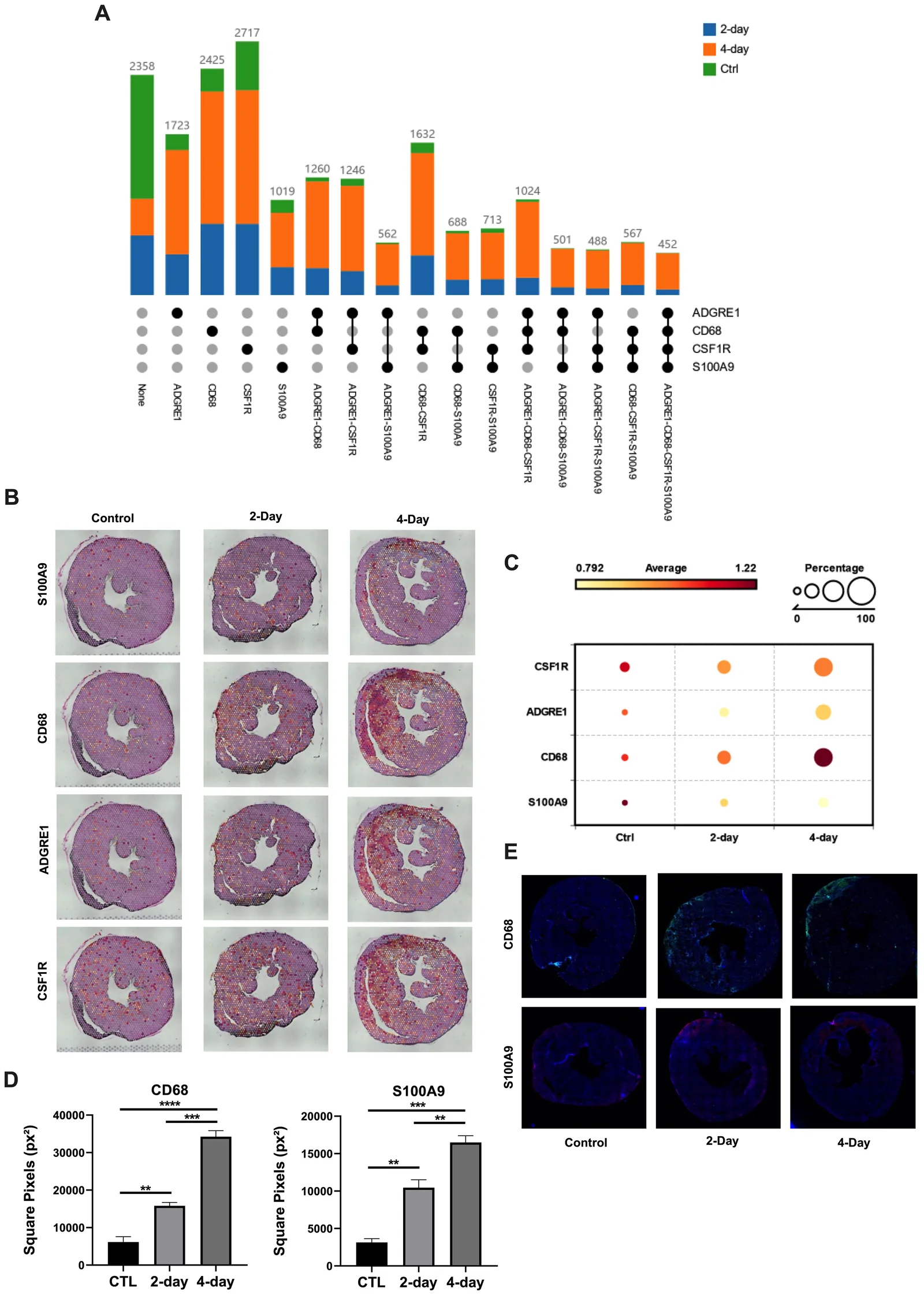
Localization of S100 expression and macrophage profile. **(A)** Intersect plot illustrating the number of cells/spots expressing both S100a9 and macrophage-associated marker genes at each selected time point during early-onset AMI compared with control samples. Because Visium spots may contain transcripts from multiple cells, co-expression was interpreted as a macrophage-associated spatial signature rather than definitive evidence of S100a9 expression by resident cardiac macrophages. **(B)** Spatial distribution and co-expression of S100a9 and macrophage genes during early-onset AMI. **(C)** Bubble heatmap of S100a9 gene expression depicting transcription level as the average -LogTPM (transcripts per million) darker color represents higher expression. Bubble size indicates percentage of cells expressing each gene (coverage of gene distribution) throughout the tissue samples. **(D)** IHC staining dor CD68 and S100a9 in infarcted heart sections. **(E)** Quantification of CD68 and S100a9. **P < 0.01, ***P < 0.001, ****P < 0.0001.

### Co-expression of fibrosis- and macrophage-related genes

During our tissue analysis, we explored the spatial relationship between macrophage-associated genes and fibrosis-associated genes during early AMI. Similar to our analysis of S100a9-associated macrophage signatures, we created intersect plots to examine co-expression of macrophage markers, including Adgre1 and Cd68, with collagen/fibrosis-associated genes, including Ccn2 and Col1a1. These fibrosis-associated spatial signatures were most evident in 2-day and 4-day tissues (**Figure 11A**). Spatially, co-localization of macrophage-associated and fibrosis-associated genes was most apparent in regions where ischemic injury could also be visualized. However, because Col1a1 and Ccn2 are also expressed by fibroblasts and myofibroblasts, these data should be interpreted as fibrosis-associated macrophage spatial signatures rather than definitive evidence that macrophages themselves express collagen-associated genes. Some studies have proposed that macrophages directly contribute collagen during cardiac scar formation (28, 29), whereas others have suggested that macrophages may transition into fibroblast-like intermediate states after myocardial injury (17, 30).

**Figure 11:**
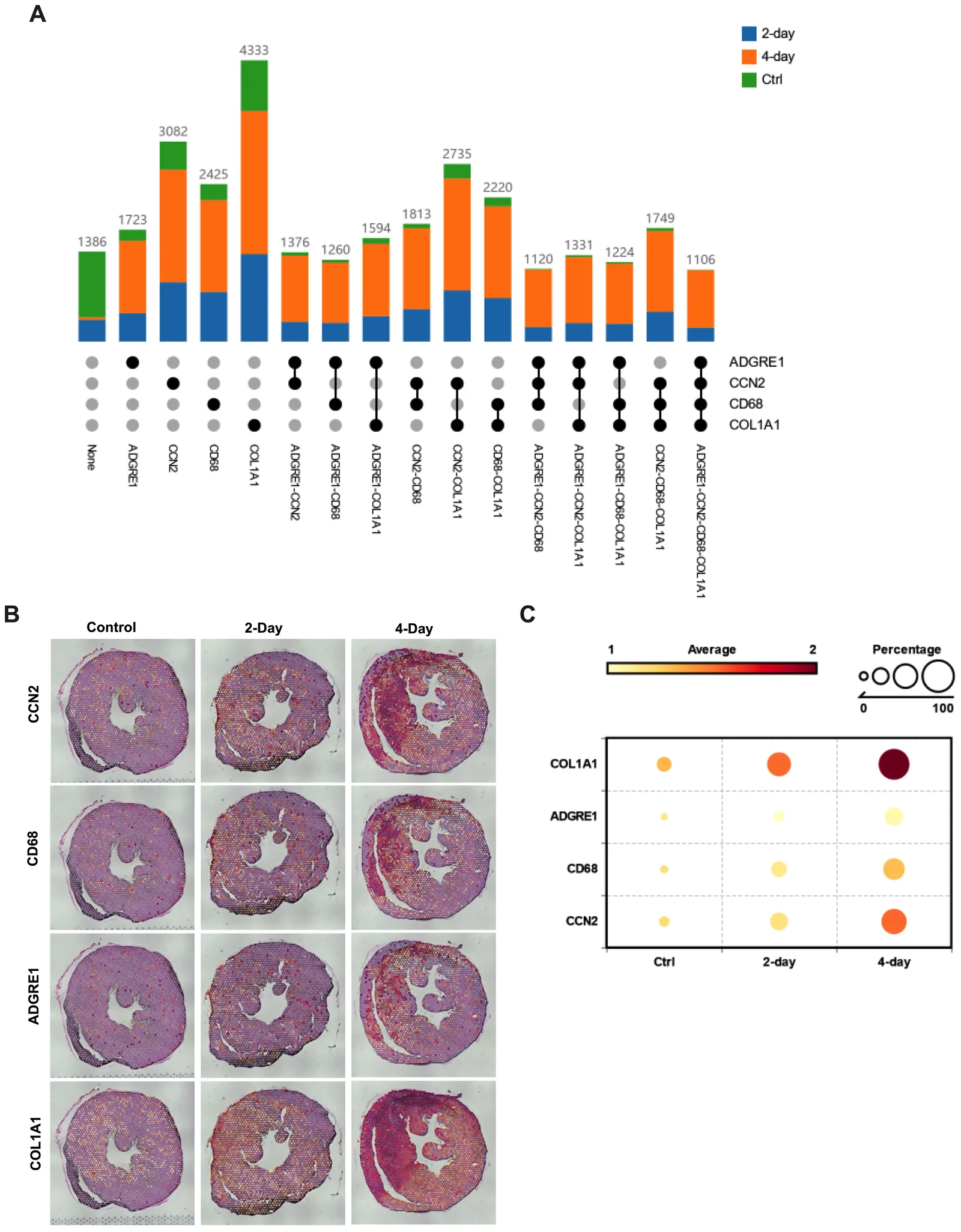
Identification of fibrogenic macrophages. **(A)** Intersect plot illustrating total number of cells/spots expressing collagen-associated genes and distinctive genes of specific macrophage subtypes and number of cells/spots at each selected time point during early-onset AMI compared to control samples. **(B)** Spatial distribution and co-expression of S100a9 and macrophage genes during early-onset AMI. **(C)** Bubble heatmap of collagen gene expression depicting transcription level as the average -LogTPM (transcripts per million) darker color represents higher expression. Bubble size indicates percentage of cells expressing each gene (coverage of gene distribution) throughout the tissue samples.

## Discussion

In this work, we describe transcriptional changes in RCM during AMI. Furthermore, we describe spatiotemporal transcriptional patterns of candidate macrophage-associated genes in cardiac tissues reflecting the phasic nature of AMI. To recapitulate a clinical representation of AMI, we used flow cytometry to isolate RCM from murine heart digests after 30 minutes of LAD ligation, followed by reperfusion for 0, 2, and 4, days. Multiplex mRNA analysis of RCM with the NanoString nCounter system provided differential expression comparisons between time points. Of the nearly 800 myeloid-specific genes probed, we identified candidate genes meeting exploratory differential-expression thresholds in a phase-dependent manner. Samples collected 2 days after AMI depicting RCM physiology during the initial “inflammatory” phase revealed upregulation of novel inflammatory genes when compared to samples collected during the ensuing “proliferative” phase. Transcription of histidine decarboxylase (HDC), for example, was upregulated in RCM collected after 2 days when compared to 4-day samples. Histidine deficiency and HDC expression have recently been identified as important processes that influence cell physiology and tissue remodeling post-AMI (31–33).

We also found enhanced expression of S100a8 and S100a9 during the inflammatory phase. S100a8 and S100a9 are calcium binding proteins termed alarmins, and it was recently determined that they are binding partners for each other. Although the effects of S100a8 and S100a9 imbalance are unclear at this time, recent publications have shown that the S100a8/S100a9 complex has dual functionality depending on the phase of AMI. S100a8/S100a9 expression contributes to MMP-9 production and extracellular matrix degradation during the inflammatory phase, while the inhibition of the complex leads to long-term pathological tissue remodeling (25–27). Immune responsive gene (Irg1) transcription was also elevated in our tissue samples following 2 days of AMI and research has shown anti-inflammatory properties of Irg1 in activated macrophages (34). Irg1 is integral to macrophage metabolism and exerts an anti-inflammatory effect by inhibiting succinate dehydrogenase (SDH) which prevents activation of the IL-1β/HIF-1α inflammatory pathway both *in vitro* and after AMI (35–38). Interestingly, differential gene expression between 2-day and 4-day samples showed Irg1 and S100a8 gene expression (but not S100a9) were also significantly increased. Moreover, elevated transcription of interferon regulatory factor-7 (Irf7) in our 4-day samples was observed, representing the “proliferative” phase of AMI compared to RCM collected after 2 days during the “inflammatory” phase. Irf7 engages multiple cellular processes, and several studies have demonstrated novel rules for Irf7 including: attenuated inflammatory cell response in swine cells, inhibition of pathological cardiac hypertrophy, mediation of tissue neovascularization, and reduction of smooth muscle cell-induced neointima formation (39–42).

Incorporation of novel spatiotemporal transcriptomic analysis was achieved with the 10X Visium platform. This allowed us to further investigate the role of macrophages in early stages of AMI. Used in combination with the BioTuring Lens software, we were able to visualize the localization of macrophage-associated transcriptional signatures during AMI according to their respective cell surface markers. The six macrophage-associated transcriptional states included RCM-associated, antigen-presenting, infiltrating, pro-inflammatory, reparative, and interferon-associated signatures. The spatial localization of macrophage heterogeneity showed that each of the subpopulations was aggregated within the infarct zone by day 4, except for the RCM population. This was especially prominent in infiltrating and M1-polarized macrophages. Cd68^+^ macrophages could also be seen localizing to the infarct zone by day 2. On the other hand, RCM exhibited highest transcription level in control samples. This was to be expected as infiltrating MDM replenish macrophages within the cardiac compartment following AMI (15). As RCM are rapidly depleted within the first hours of infarction, it is possible that what we observe in our 2-day samples is proliferation of RCM after an initial wave of rapid depletion. Direct assessment of RCM proliferation using Ki67, EdU, or BrdU labeling was not performed in the present study; therefore, this interpretation remains inferential and requires future validation.

A recent study using 10x Genomics Visium spatial transcriptomics has shown that this platform is useful for mapping inflammatory, stromal, and tissue-remodeling programs within injured organs while preserving anatomical context (43). This study also emphasizes an important limitation of Visium-based analysis: each capture spot may contain transcripts from more than one cell. Therefore, spatial transcriptomic data are most appropriately interpreted as tissue-localized transcriptional signatures rather than definitive single-cell identities unless supported by deconvolution, single-cell reference mapping, RNAscope, or multiplex immunofluorescence. In the present study, we therefore interpret the Visium results as macrophage-associated spatial signatures that suggest localization of resident, infiltrating, inflammatory, reparative, interferon-associated, and fibrosis-associated macrophage programs during early AMI, rather than as proof of discrete macrophage subtypes at single-cell resolution.

We also observed early recruitment of M2 macrophages expressing Fabp5 and Timp2 to the injury site which is indicative of the diverse role played by different macrophage populations during AMI. Interestingly, spatial analysis revealed a delayed tissue response of specific genes associated with AMI compared to gene transcription by RCM *ex vivo*. For example, comparison of S100a9 showed limited gene expression in tissues after 2 days during the “inflammatory” phase and robust tissue expression by day 4 during the “proliferative” phase. Co-expression of S100a9 transcripts with macrophage-associated markers indicated that macrophage-rich regions contribute substantially to the S100a9 spatial signal. However, these data do not establish that resident cardiac macrophages are the primary cellular source of S100a9. Given the established association of S100A9 with inflammatory myeloid responses, the observed temporal and spatial pattern identifies S100a9-associated signaling as a candidate inflammatory pathway during early AMI. Taken together, our data support an association between early RCM-enriched transcriptional responses and subsequent macrophage-associated inflammatory signals within injured cardiac tissue.

An important limitation of the RCM-enriched NanoString analysis is that low-level contribution from other myeloid populations cannot be completely excluded. The detection of Elane transcription provides evidence that neutrophil-derived transcripts may contribute to some inflammatory signals in the sorted-cell dataset. Neutrophils are known to express S100a8 and S100a9 in the setting of inflammation. Therefore, the contribution of S100a8 and S100a9 transcripts from cells other than RCM cannot be determined from the present NanoString dataset. Although the gating strategy was designed to enrich RCM and exclude non-RCM populations, low-level contamination by neutrophils or CCR2-positive myeloid cells cannot be fully excluded and may contribute to inflammatory transcripts such as S100a8 and S100a9. Ccr2 expression was also detected in the sorted-cell dataset. Although the flow-cytometric analysis indicated that CCR2-positive cells represented a relatively small population, their contribution to the pooled transcriptional signal cannot be excluded (**Figure 1A**). It has been observed that 2 of the 4 RCM phenotypes express Ccr2 and are distinguished as generally being either MHCII^Lo^ or MHCII^Hi^ while exhibiting much of the same behavior as their Ccr2^-^ counterparts (4, 44, 45). In addition, only a limited number of RCM can be collected given their scarcity in the tissues. However, transcription of genes representative of cell types other than RCM (including Elane and Ccr2) were all statistically low, while the expected transcriptional profile of RCM markers like Timd4, Mafb, and Igf1 were among the highest expressed genes (**Supplementary Materials 2 Table 1**). It is certainly possible that contamination from neighboring cells affected Visium outputs. However, we intentionally used multiple established macrophage markers to mitigate this effect in our analysis, and utilized combinations of cell surface markers that would exclude most other cells.

Several limitations should be considered when interpreting the S100a8/S100a9 findings. Because the NanoString analysis was performed on pooled RCM-enriched samples obtained after ischemia/reperfusion injury, contributions from infiltrating monocyte-derived macrophages or low-level contamination by neutrophils cannot be completely excluded. The detection of inflammatory transcripts, including Elane, further supports the need for caution when assigning these signals specifically to resident cardiac macrophages. In addition, although S100A9 protein localization was assessed by immunostaining and showed spatial association with macrophage-rich regions, the present study did not directly test S100A9 function through selective inhibition, genetic manipulation, or rescue experiments. Therefore, our findings support a temporal and spatial association of S100a8/S100a9 with macrophage-associated inflammatory responses rather than a causal role for S100A9 in AMI progression. Future studies combining cell-specific molecular validation with functional S100A9 perturbation will be required to determine the cellular source and causal contribution of S100A9 during IRI.

Our fibrosis-associated macrophage findings should also be interpreted in the context of previous reports describing macrophage contributions to scar formation and fibroblast-like macrophage states after cardiac injury. Prior studies have reported dynamic macrophage transcriptional programs after myocardial injury, macrophage-to-fibroblast-like transitions during infarct healing, and macrophage-associated collagen deposition during cardiac repair (17, 28–30). In agreement with these reports, our Visium analysis showed spatial co-localization of macrophage-associated markers, including Cd68 and Adgre1, with fibrosis-associated genes such as Col1a1 and Ccn2 in injured regions. However, because Col1a1 and Ccn2 are also highly expressed by fibroblasts and myofibroblasts, and because each Visium spot may contain multiple cells, these data do not prove that macrophages themselves express collagen-associated genes. These signatures may represent fibrosis-like macrophage states, macrophage-fibroblast proximity, or mixed-cell Visium spots within the infarct region. Future validation by RNAscope, multiplex immunofluorescence, or single-cell/single-nucleus RNA sequencing will be required to determine whether these fibrosis-associated transcripts are expressed by macrophages directly.

In summary, we used complementary transcriptional approaches to characterize RCM-associated and macrophage-associated spatial signatures during the early phases of AMI. NanoString profiling of pooled RCM samples identified candidate inflammatory, reparative, and extracellular matrix-associated transcriptional programs, while Visium spatial transcriptomics localized macrophage-associated signatures within injured cardiac tissue. These findings support a model in which early RCM-associated transcriptional changes and later infarct-zone macrophage-associated signatures contribute to the dynamic immune landscape after AMI. Future studies using cell-specific molecular validation, S100A9 functional perturbation, single-cell or single-nucleus transcriptomics, RNAscope, and multiplex immunofluorescence will be required to define the cellular sources and causal roles of these candidate pathways and to determine their therapeutic relevance.

## Data Availability

The datasets presented in this study can be found in online repositories. The names of the repository/repositories and accession number(s) can be found below: GSE333790 (GEO) and GSE346811.
