# Supplementary material for: Spatiotemporal transcriptomic signatures of resident cardiac macrophages and macrophage-associated spatial states during acute myocardial infarction
Source: Front Immunol. 2026 Oct 20;17:1882796. doi: 10.3389/fimmu.2026.1882796 (PMC13727469; doi:10.3389/fimmu.2026.1882796)
Supplement: Supplementary file 1 [file DataSheet1.pdf]

frontiers

The International Society for the Advancement of Cytometry (ISAC) has highlighted the importance of including comprehensive methodological information to ensure data reproducibility and reliability. In line with this, Frontiers in Immunology now requires authors to submit a checklist for manuscripts that involve flow or mass cytometry. This checklist helps standardize the reporting process, improving the quality and transparency of published data. By doing so, we support scientific progress, making it easier for other researchers to replicate and validate experiments.

This form should be submitted with any manuscripts using flow or mass cytometry.

Sample/specimen/material description

- ☐ Total blood
- ☐ PBMCs
- ☑ Organ digests

Other____

Did the samples suffer any treatment before or after incubation with the antibodies?

- ☐ Drug ____________________________________
- ☐ Cell permeabilization ______________________
- ☐ Dye _____________________________________
- ☑ Propidium iodine
- ☐ Not applicable

Other ____

Instrument and antibodies

Name of the Cytometer FACSAria___________________________________

Antibodies and targets	Fluorochrome/ Metal	Catalog number/Company
e.g. anti-CD4	FITC	Cat. XXX/ XXX ltd.
Anti-CD64	BV421	139309/BioLegend

Anti-MHC II	Super Bright 645	64-5321-82/Thermo
Anti-CD45	FiTC	103107/BioLegend
Anti-CCR2	Horizon BB700	747965/BD Biosciences
Anti-LYVE1	PE-Cy7	25-0443-82/Thermo
Anti-TIMD4	PE	130006/BioLegend
Anti-CD11b	APC-Cy7	101226/BioLegend

Data analyses

1. Name of the software FlowJo______________________________
2. Reference gating strategy in the manuscript or supplementary material

Gating strategy in (eg Figure X) Figure 1_____________________________
