# Supplementary material for: Spatiotemporal transcriptomic signatures of resident cardiac macrophages and macrophage-associated spatial states during acute myocardial infarction
Source: Front Immunol. 2026 Oct 20;17:1882796. doi: 10.3389/fimmu.2026.1882796 (PMC13727469; doi:10.3389/fimmu.2026.1882796)
Supplement: Supplementary file 2 [file Table2.docx]

**Supplementary figure 1: A)** Full gene list of nCounter® Mouse Myeloid Innate Immunity Panel V2 (double-click to see full list and details). **B)** Expression heatmap of monocyte and macrophage genes to identify and track spatiotemporal transcription of these cell types. Values are represented by color and average Log1p(TPM) expression. Coverage over the sample is represented by the relative size of the dot in the heatmap. **C)** Cell-surface markers used to distinguish macrophage subtypes.

**Supplementary Material Table 1**

All genes identified using the 10x Visium platform (double-click to expand).
